# First international diagnostic accuracy study for the serological detection of West Nile virus infection

**DOI:** 10.1186/1471-2334-7-72

**Published:** 2007-07-03

**Authors:** Matthias Niedrig, Oliver Donoso Mantke, Doris Altmann, Hervé Zeller

**Affiliations:** 1 Centre for Biological Safety (ZBS-1), Robert Koch-Institut, Nordufer 20, D-13353 Berlin, Germany; 2 Department of Infectious Disease Epidemiology (FG 31), Robert Koch-Institut, Seestraße 10, D-13353 Berlin, Germany; 3 Unit Biologie des Infections Virales Emergentes (UBIVE), Institut Pasteur, 21 Avenue Tony Garnier, 69365 Lyon cedex 7, France

## Abstract

**Background:**

The diagnosis of an acute or convalescent West Nile (WN) virus infection can be confirmed by various serological assays such as enzyme immunoassay (EIA), immunofluorescence assay (IFA), or neutralisation test (NT) which are conducted by a growing number of laboratories. However, as the degree of proficiency may vary between laboratories, quality control measures for laboratory diagnostics are essential.

**Methods:**

We have performed an external quality assurance (EQA) programme for the serological detection of WN virus infection to assess the diagnostic quality of laboratories. The participating laboratories received a proficiency panel of 10 coded lyophilised test samples comprising four antisera positive for WN antibodies as positive controls, three antisera positive for antibodies against other heterologous flaviviruses plus one multireactive unspecific serum as specificity controls, and two negative serum samples.

**Results:**

Twenty-seven laboratories from 20 different countries in Europe, the Middle East, the Americas and Africa participated in this EQA programme. Applying the proficiency criteria of this study, only eight laboratories correctly analysed all samples with their respective EIA, IFA or NT methods. Eighteen laboratories correctly identified between 77.8 and 90% of the samples, and one laboratory identified only 70% correctly with a clear need to eliminate cross-reactivity with other antisera, particularly those elicited by yellow fever virus. Differentiation between the results for IgM and IgG was considered separately and revealed that IgM-antibodies were detected less frequently than IgG-antibodies (p < 0.001). However, the assay used was not a significant technical factor influencing laboratory performance.

**Conclusion:**

The EQA programme provides information on the quality of different serological assays used by the participating laboratories and indicates that most need to improve their assays, in particular to avoid cross-reactions with antibodies to heterologous flaviviruses.

## Background

West Nile (WN) virus is a mosquito-transmitted flavivirus which belongs to the Japanese encephalitis virus group. It occurs throughout Africa, the Middle East, southern Europe, Russia, India and Indonesia, and was recently introduced into North America [1-3]. Migratory birds are involved in the transmission cycle of this virus as amplifying hosts, and humans and horses are considered to be accidental dead-end hosts [1,4]. In humans, the majority of WN virus infections cause a non-symptomatic or a mild flu-like illness. However, some infections can cause encephalitis which may lead to death, particularly in elderly patients [3]. WN virus is a clear example of the tremendous impact that virus spread and evolution can have on human beings. From 1999 to 2006 there were 8422 neuroinvasive WN cases (including 889 fatalities) reported in the United States [5]. The incidence of WN virus in Europe is comparatively poorly studied and the risk for a similar epidemic, although low, cannot be precisely estimated [6]. The availability of reliable serological assays such as the enzyme immunoassay (EIA), immunofluorescence assay (IFA) or neutralisation test (NT) is an important prerequisite for the clinical diagnosis and epidemiological surveillance of WN virus infections.

A major problem for WN serological assays is their high degree of cross-reactivity with antibodies produced in response to other simultaneous and/or previous flavivirus infections [7]. False positive results are due to the cross-reactivity of antibodies specific for related epitopes found on other flaviviruses (e.g. Saint Louis encephalitis-, dengue-, yellow fever-, tick-borne encephalitis-, or Japanese encephalitis virus) induced by natural infection or vaccination. This is mainly true for IgG- and in some cases for IgM-antibodies. Since differentiation of a specific immune response is difficult, a fourfold increase in antibody titre in follow-up patient sera is mandatory for a positive diagnosis [8].

Several research laboratories have developed serological assays for WN virus infection and a number of commercial test kits are now available [9]. However, the performance of these methods varies considerably between laboratories. Comprehensive external quality control studies for WN serology have not yet been performed and little information is available about the relative and overall proficiency in different laboratories. Comparative testing of well-characterised samples is the best method of identifying weaknesses of single laboratories or of certain methodological components. The aim of this study was to assess the diagnostic accuracy across participating laboratories and the tests they use by performing the first international external quality assurance (EQA) study for the serological detection of WN virus infection.

## Methods

### Participants and recruitment

Twenty-seven laboratories from 20 different countries participated in this EQA programme, including 20 laboratories from Europe, three from the Middle East, three from North or South America and one from Africa. A complete list of participants is given in the acknowledgements section. The study was announced as an EQA study on diagnostic proficiency run by the European Network for diagnostics of 'Imported' Viral Diseases (ENIVD), including publication of the results in a comparative and anonymous manner. Participation was open and free of charge to all laboratories performing WN diagnostics. Selection of invitees was based on the register of ENIVD members as well as on their contributions to the literature relevant to this topic.

### Preparation of test samples

Test samples for the proficiency panel were generated by diluting well-characterised human sera with fresh-frozen plasma tested and confirmed to be negative for HIV, hepatitis B-, hepatitis C-, WN- and non-WN-flaviviruses. After dilution, the enriched serum samples were heat-treated (56°C, 1 h), frozen and lyophilised in aliquots of 100 μl to prepare proficiency panels consisting of 10 test samples. As positive controls the panel comprised aliquots of four antisera positive for WN antibodies purchased from SeraCare Life Sciences, Milford, MA, USA. For specificity controls, aliquots of three antisera containing antibodies reactive with heterologous flaviviruses (tick-borne encephalitis-, yellow fever- and Dengue virus) provided by reference laboratories of our network and one serum with a known unspecific reactivity against cell and mitochondrial structures provided by EUROIMMUN AG, Lübeck, Germany were included. Two additional aliquots from confirmed seronegative samples served as negative controls. Two sets of this EQA panel were tested for specific activity by two expert laboratories to confirm the quality of the samples after preparation.

### Distribution of test samples and given instructions

All samples were sent out by regular mail and arrived within 1 week after sending, according to the date of arrival provided by the participants. It was recommended that the samples be resuspended with 100 μl distilled water and centrifuged for 5 min to remove any aggregates before testing. The participants were asked to analyse the material by the diagnostic methods they routinely used for the serological detection of WN virus infection. There was no obligation concerning the test procedure to be used but information concerning the type/format (EIA, IFA, NT) and whether it was an in-house assay or a commercial kit was requested. No further information regarding the detailed processing of the NTs (format, cell line used, 50% or 90% calculation, reference etc.) were provided by the participating laboratories.

### Evaluation of participants' results

The following two criteria were selected as the minimum requirements for successful overall proficiency, scored with 10 points (= 100%). First, laboratories had to detect the four WN-positive samples irrespective of the differentiation between IgM and IgG; this means that at least one of these tests had to give a positive result. Second, the antisera containing cross-reactive antibodies to heterologous flaviviruses (tick-borne encephalitis-, yellow fever- and dengue virus) or the known unspecific serum should not give a positive result and/or should be recognised as being unspecific. Equivocal or borderline results with the non-WN-flavivirus positive samples were treated as negative. The indeterminate result of Lab. No. 15 in one of the negative samples was identified as such and was not used in the evaluation. False positive or negative results as well as non-clarified results with clinical consequences were given a score of -1. The differentiation between IgM and/or IgG results was considered separately and gave additional information concerning the quality of the laboratory diagnostics. Data collected were entered into Microsoft Excel (Microsoft Corp., Bellingham, WA, USA) and analysed using SPSS 14.0 for Windows. Results with respect to categorised variables were analysed by the chi-square test. Whether or not common technical factors or particular samples influenced the performance of the participating laboratories was assessed by univariate and multivariate logistic regression. A p-value < 0.05 was considered to indicate statistical significance.

## Results and Discussion

### Overall proficiency of the participants

Table 1 summarises the results obtained and gives details on the serological assays used by the participating laboratories. Only eight (~30%) of 27 participating laboratories passed the minimum requirements for successful performance. The main problem of the other laboratories in achieving the minimum proficiency standards were due to cross-reactivities with sera containing antibodies to heterologous flaviviruses, mainly with a serum from a yellow fever vaccinee (14 laboratories; ~52%). Surprisingly, this sample #7 exhibited cross-reactions in three out of seven in-house NTs performed by the participants (Table 1). This demonstrates the limitation of this highly specific assay and the great variation it could cause in the overall proficiency, as described previously [10]. However, the NT is not a suitable assay for rapid diagnosis and should be combined with an initial detection method. The distinct cross-reactivity with sera positive for antibodies against yellow fever virus is a well known difficulty for all common serological assays and is also mentioned in the instruction leaflet of the commercial test kits [11]. Poor performance was also attributable to a lack of sensitivity in five laboratories (Lab. No. 9, 10, 24, 30, 39) while in two other laboratories (Lab. No. 11, 17) it was caused by false positive results, possibly resulting from inadequate internal controls (Table 1).

**Table 1:** Overall results of the EQA for the serological detection of WN virus infection

		Sample N°										
			
Lab. N°	Assay used	#8 anti-WN	#12 anti-WN	#5 anti-WN	#6 anti-WN	#10 anti-TBE	#7 anti-YF	#11 anti-DEN	#3 neg.	#2 neg.	#1 unspec.	Correct results in %
2^‡^	EIA/IFA_ih_	(-)/+	(-)/+	(-)/+	-/+	-	-	-	-	-	-	100.0
4	EIA_ih_	+/+	+/+	+/+	-/+	-*	-*	-	-	-	-	100.0
8	EIA_ih_	+/(-)	+/+	+/+	-/+	-	**-/**e	-	-	-	-	100.0
18	EIA_ih_	+/+	+/+	+/+	-/+	-	-*	-	-	-	-	100.0
29	IFA_ih_	(-)/+	(-)/+	+/+	-/+	-	-*	-	-	-	-	100.0
37	EIA_ih_	+	+	+	+	-	-*	-	-	-	-	100.0
5	**EIA**_F_	+/+	e/+	+/+	-/+	-	-/**(+)**	-	-	-	-	90.0
6	IFA_ih_	(-)/+	(-)/+	+/+	-/+	-	-/**(+)**	-	-	-	-	90.0
9	**IFA**_P_	**(-)**	(+)/(+)	+/+	-/(+)	-	-*	-	-	-	-	90.0
10	IFAi_h_	(-)/+	**(-)**	(-)/+	-/+	-	-	-	-	-	-	90.0
14	EIA_ih_	(-)/+	+/+	(-)/+	-/+	-	-/**(+)**	-	-	-	-	90.0
16	**EIA**_F_	+/+	e/+	+/+	-/+	-	-/**(+)**	-	-	-	-	90.0
17	IFA_ih_	(-)/+	(-)/+	+/+	-/+	-	-/e	-	-	-	-/**(+)**	90.0
20	**EIA**_F_	+/+	e/+	+/+	-/+	-	-/**(+)**	-	-	-	-	90.0
23	**EIA**_F_	(-)/+	(-)/+	(-)/+	-/+	-	-/**(+)**	-	-	-	-	90.0
35	**EIA**_F+P_	e/+	e/+	e/+	-/+	-	-/**(+)**	-	-	-	-	90.0
11	IFA_ih_	+/+	+/+	+/+	(+)/+	-	-/**(+)**	-	-	-	**(+)**	80.0
13	**EIA**_F_	+/+	e/+	e/+	-/+	-	-/**(+)**	**(+)/-**	-	-	-	80.0
24	EIAi_h_	+	**(-)**	+	**(-)**	-	-	-	-	-	-	80.0
25	**EIA**_F_	(+)/+	(-)/**e**	(+)/+	-/+	-	-/**(+)**	-	-	-	-	80.0
33	EIA_ih_	+	+	+	+	**(+)**	**(+)**	-	-	-	-	80.0
15	**EIA**_F_	+/+	+/+	+/+	-/+	**(+)/-**	-/**(+)**	ind.	-	-	-	77.8
30	EIA_ih_	**(-)**	**(-)**	+	+	-	**(+)**	-	-	-	-	70.0
7	NT_ih_	(+)	(+)	+	+	-	-	-	-	-	-	100.0
36	NT_ih_	e	+	+	+	-	-	-	-	-	-	100.0
11	NT_ih_	+	+	+	(+)	-	-	-	-	-	**(+)**	90.0
5	NT_ih_	+	+	+	+	-	**(+)**	**(+)**	-	-	-	80.0
12	NT_ih_	+	+	+	+	**(+)**	**(+)**	-	-	-	-	80.0
39	NT_ih_	**(-)**	**(-)**	(+)	(+)	-	-	-	-	-	-	80.0
30	NT_ih_	**(-)**	**(-)**	+	+	-	**(+)**	-	-	-	-	70.0

WN = West Nile; TBE = tick-borne encephalitis; YF = yellow fever; DEN = dengue; neg. = negative; unspec. = unspecific. x/x = IgM/IgG result: - = correct negative result; (-) = false negative result without meaning; + = correct positive result; (+) = weak positive result; e = equivocal result; ind. = indetermined. -* = negative for WN virus, but other flavivirus detected; -^# ^= negative for WN virus, but unspecific reaction detected. Results marked in bold mean false negative/positive results or non conclusive results which may have consequences for the decision of the clinician. ^‡ ^Laboratory reported combined results: IgM tested by EIA and IgG tested by IFA. In other cases laboratories have reported combined results for IgM and IgG (single results).

EIA: Enzyme immunoassay; IFA: Immunofluorescence assay; NT: neutralisation test. Assays marked with the index "ih" are in-house assays. Assays marked in bold are commercial assays. Manufacturers: F = Focus Diagnostics Inc., Cypress, CA; USA, P = PanBio Ltd., Queensland, Australia.

### Sensitivity for WN specific IgM and IgG antibodies

Of the four clearly WN positive samples, samples #5 and #6 were identified by nearly all laboratories in all assays (100% and 96.3%, respectively) whereas samples #8 and #12 were detected in 88.8% and 81.5% of all analyses performed (Table 1). Interestingly, the detection of IgM antibodies did not correlate with the sensitivity for detecting IgG antibodies (Table 2). In those laboratories (N = 19) analysing both the specific IgM and IgG responses, anti-WN specific IgM activity was correctly identified for sample #6 by 94.7% (correct negative results), for #5 by 78.9%, for #12 by only 63.2% and #8 by only 57.9% (all correct positive results). IgM antibodies were detected significantly less (p < 0.001) than IgG antibodies with correct test results between 89.5 and 100% (correct positive results). The results clearly demonstrate that either the assays have to be improved or that the assays were not performed correctly. As described in previous EQA studies [12], these findings indicate that there is a considerable risk that acute infections are overlooked. Reliable assays for IgM detection are a prerequisite for the diagnosis of acute or recent infections in humans and their development is therefore very important.

**Table 2:** Evaluation of the WN positive samples in the test panel*

Sample	Expected result IgM/IgG	Fraction of correct classified results for IgM in %	Fraction of correct classified results for IgG in %
#8 anti-WN	+/+	57.9 (11)	89.5 (17)
#12 anti-WN	+/+	63.2 (12)	89.5 (17)
#5 anti-WN	+/+	78.9 (15)	100.0 (19)
#6 anti-WN	-/+	94.7 (18)	100.0 (19)

**overall:**		**73.7**^†^	**94.7**

* No. of laboratories: 19. Number of laboratories with correct results shown in brackets.

^† ^IgM-antibodies are less detected than IgG-antibodies (Χ^2 ^= 12.7; p < 0.001).

### Factors influencing laboratory performance

Nine laboratories used commercial tests (8× West Nile virus ELISA IgG/IgM test, Focus Diagnostics Inc., Cypress, CA, USA; 1× West Nile virus ELISA IgG/IgM test, PanBio; 1× West Nile virus IFA kit, PanBio Ltd., Queensland, Australia) and 18 laboratories used in-house tests (9× EIA; 6× IFA and 7× NT). We therefore assessed whether common technical factors influenced the accuracy of the participating laboratories. While we found no significant variation (data not shown) when comparing the two different technical factors, i.e. assay type/format (EIA vs. IFA vs. NT) and assay origin (in-house assay vs. commercial assay), multivariate logistic regression analysis revealed that the samples used had a significant influence on laboratory performance (Table 3). Sample #7 showed – compared to the other samples (except #2, #3 and #5) – a 5- to 33-fold lower likelihood of correct classification. This result underlined with statistical significance the analysis of overall proficiency. The assays tested clearly need to eliminate cross-reactivity with antisera positive for related antibodies, particularly those specific for yellow fever virus. This will be a future objective for the improvement and development of diagnostic methods for WN virus. As previously mentioned and in agreement with a previous EQA study for the serological detection of dengue virus infection [12,13], there was no significant difference between the use of commercial or in-house assays.

**Table 3:** Samples influencing laboratory performance*

Sample	p-value	Odds-ratio	95% confidence interval
overall	0.001		
7		1	
1	0.001	10.3	2.6 – 41.4
6	0.001	33.1	4.0 – 275.7
8	0.002	7.4	2.1 – 26.6
10	0.001	10.3	2.6 – 41.4
11	0.001	15.4	3.1 – 76.8
12	0.009	4.6	1.5 – 14.4

* Multivariate logistic regression analyses. All samples shown had a significant influence on performance (see text for details).

## Conclusion

This study demonstrates the importance of quality control measures in the serological detection of WN virus infection. The results clearly indicate a need for certain laboratories to improve their tests, e.g. to avoid cross-reactivity with sera containing antibodies specific for heterologous flaviviruses. Comparative testing of well-characterised samples provided all participating laboratories with the opportunity to identify their weaknesses and to improve their methodologies, which should then be confirmed in subsequent studies. However, it is important that new improved assays with a higher specificity will be developed that can discriminate between antibody responses to different flavivirus infections by including measurements of antibody avidity.

## List of abbreviations

EIA Enzyme immunoassay

ENIVD European Network for diagnostics of 'Imported' Viral Diseases

EQA External quality assurance

HIV Human immunodeficiency virus

IFA Immunofluorescence assay

NT Neutralisation test

WN West Nile

## Competing interests

The author(s) declare that they have no competing interests.

## Authors' contributions

MN designed the study together with HZ and was responsible for data acquisition. MN and ODM were responsible for evaluation and interpretation of data and wrote the manuscript. DA provided statistical analysis. HZ provided review and expert advice. All authors read and approved the final manuscript.

## Pre-publication history

The pre-publication history for this paper can be accessed here:


